# *N*-3-oxo-octanoyl-homoserine lactone-mediated priming of resistance to *Pseudomonas syringae* requires the salicylic acid signaling pathway in *Arabidopsis thaliana*

**DOI:** 10.1186/s12870-019-2228-6

**Published:** 2020-01-28

**Authors:** Fang Liu, Qian Zhao, Zhenhua Jia, Cong Song, Yali Huang, Hong Ma, Shuishan Song

**Affiliations:** 1 Biology Institute, Hebei Academy of Sciences, 46th South Street of Friendship, Shijiazhuang, 050051 China; 2 Hebei Engineering and Technology Center of Microbiological Control on Main Crop Disease, 46th South Street of Friendship, Shijiazhuang, 050051 China

**Keywords:** *Arabidopsis thaliana*, Quorum sensing (QS), Priming, Salicylic acid (SA), Plant defense, *N*-3-oxo-octanoyl-homoserine lactone (3OC8-HSL)

## Abstract

**Backgroud:**

Many Gram-negative bacteria use *N*-acyl-homoserine lactones (AHLs) to communicate each other and to coordinate their collective behaviors. Recently, accumulating evidence shows that host plants are able to sense and respond to bacterial AHLs. Once primed, plants are in an altered state that enables plant cells to more quickly and/or strongly respond to subsequent pathogen infection or abiotic stress.

**Results:**

In this study, we report that pretreatment with *N*-3-oxo-octanoyl-homoserine lactone (3OC8-HSL) confers resistance against the pathogenic bacterium *Pseudomonas syringae* pv. *tomato* DC3000 (*Pst*DC3000) in Arabidopsis. Pretreatment with 3OC8-HSL and subsequent pathogen invasion triggered an augmented burst of hydrogen peroxide, salicylic acid accumulation, and fortified expression of the pathogenesis-related genes *PR1* and *PR5*. Upon *Pst*DC3000 challenge, plants treated with 3OC8-HSL showed increased activities of defense-related enzymes including peroxidase, catalase, phenylalanine ammonialyase, and superoxide dismutase. In addition, the 3OC8-HSL-primed resistance to *Pst*DC3000 in wild-type plants was impaired in plants expressing the bacterial *NahG* gene and in the *npr1* mutant. Moreover, the expression levels of isochorismate synthases (*ICS1*), a critical salicylic acid biosynthesis enzyme, and two regulators of its expression, *SARD1* and *CBP60g*, were potentiated by 3OC8-HSL pretreatment followed by pathogen inoculation.

**Conclusions:**

Our data indicate that 3OC8-HSL primes the Arabidopsis defense response upon hemibiotrophic bacterial infection and that 3OC8-HSL-primed resistance is dependent on the SA signaling pathway. These findings may help establish a novel strategy for the control of plant disease.

## Background

The co-evolution of plants and microorganisms has allowed plants to develop sophisticated pathogen defense mechanisms. Pathogen detection in plants involves the recognition of pathogen- or microbe-associated molecular patterns (PAMPs or MAMPs) [1, 2] by pattern recognition receptors (PRRs) and the activation of pattern-triggered immunity (PTI). Although pathogens may secrete PTI-inhibiting effectors into plant cells through the type III secretion system (TTSS), plants, in turn, activate a second layer of defense called effector-triggered immunity (ETI), which is activated upon the recognition of pathogen effector proteins in the host cytoplasm or apoplast [3, 4]. In addition to the local defense mechanisms of PTI and ETI, plant tissues distal to infection sites can induce systemic resistance. The two best characterized mechanisms of systemic resistance are induced systemic resistance (ISR) and systemic acquired resistance (SAR); the former responds to beneficial soil-borne microbes, while the latter is associated with pathogen attack [5–8]. Resistance mechanisms can also be stimulated by chemical treatments, including 2.6-dichloro-isonicotinic acid (INA), benzo-(1, 2, 3)-thiadiazole-7-carbotionic acid S-methyl ester (BTH), and ß-amiobutyric acid (BABA) [9, 10]. INA and BTH are analogs of the plant hormone SA. Several induced resistance processes are associated with the phenomenon of priming, which is an enhanced capacity to express specific defense responses upon pathogen attack [9]. Priming enables cells to respond to much lower levels of stimulus in a more rapid and robust manner compared to non-primed cells [9, 11]. As such, primed plants are in a physiological state of readiness to rapidly and strongly defend against pathogen challenge following an initial stimulus; this state potentially minimizes the associated metabolic costs insofar as the metabolic requirement of the priming mechanism itself is relatively low [11–14]. The priming phenomenon was first reported by Kauss and colleagues [15], and since then, several priming inducers have been documented. BABA-induced priming functions through a salicylic acid (SA) and abscisic acid (ABA)-dependent pathway, and it induces enhanced callose deposition and salt stress tolerance [16]. BABA-induced resistance also interferes with the action of the bacterial toxin coronatine produced by the pathogen *Pseudomonas syringae* [17]. Azelaic acid was characterized as a mobile metabolite that can accumulate in local and systemic tissues upon SAR to prime plants for enhanced SA production, and it confers resistance to *P. syringae* [18]. In addition to pathogen-derived elicitors, a number of low-molecular-weight metabolites including methyl salicylate (MeSA), dehydroabietinal, azelaic acid, pipecolic acid, and β-aminobutyric acid may be involved in the induction of systemic resistance in plants [18–20]. Furthermore, treatment with SA at a low concentration or its commercial derivative BTH, was shown to condition a defense reaction that led to a faster plant response upon pathogen attack [21]. These data suggest that disease resistance triggered by priming inducers could serve as a basis for novel disease control strategies and contribute to the development of sustainable agriculture.

*N*-acyl-homoserine lactones (AHLs) belong to a class of bacterial quorum sensing (QS) signals used for bacterial cell-to-cell communication. Many Gram-negative bacteria, both beneficial and pathogenic, produce AHLs and use them to coordinate the behavior of individual cells within a population. Several reports have suggested that AHLs can elicit an immune modulatory response in a broad range of mammalian cell lines; both stimulatory and suppressant immune effects were observed depending on AHL concentration and cell type [22–25]. Jahoor et al. (2008) demonstrated that the peroxisome proliferator-activated receptors PPARr and PPARβ, members of the nuclear hormone receptor (NHR) family, may be candidate AHL receptors in animals. AHLs are known to be perceived by plant cells, which in turn specifically respond to these bacterial signals [25]. Proteomic and transcriptomic analyses have shown that plant roots respond to AHLs with significant changes in expression levels [26–29]. Other studies have shown that AHLs can regulate plant root architecture in a structure- and dosage-dependent manner [27, 30]. G-protein and calcium signaling have also been implicated in the responses of plants to bacterial AHLs [31–33]. Previously, we reported that AtMYB44 positively regulated the induction of primary root elongation in Arabidopsis by *N*-3-oxo-hexanoyl-homoserine lactone (3OC6-HSL) [34]. In tomato, colonization of the root surface with *Serratia liquefaciens* MG1, which produces *N*-butyl-homoserine lactone (C4-HSL) and *N*-hexanoyl-homoserine lactone (C6-HSL), induces systemic resistance against the leaf-pathogenic fungus *Alternaria alternata*, whereas the AHL-negative *S. liquefaciens* mutant MG44 fails to induce such resistance [29]. Likewise, inoculation with *Serratia plymuthica* HRO-48, which produces C4−/C6-HSL, *N*-3-hydroxy-butyl-homoserine lactone, and *N*-3-hydroxy-hexanoyl-homoserine lactone, was found to protect cucumber from the damping-off disease caused by *Pythium aphanidermatum* and tomato and bean from *Botrytis cinerea* infection; in contrast, an *S. plymuthica* mutant with deficient AHL production could not protect against these pathogens [35]. Zarkani et al. (2013) showed that resistance against *Pseudomonas syringae* induced by *Ensifer meliloti* (*Sinorhizobium meliloti*) in Arabidopsis plants depended on the accumulation of *N*-3-oxo-tetradecanoyl-homoserine lactone (3OC14-HSL), whereas inoculation with an AHL-negative *S. meliloti* strain or a 3OC8-HSL-producing *Rhizobium etli* strain had no impact on resistance against *P. syringae* [36]. Another report described the resistance-inducing effects of *S. meliloti* on crop plants such as barley, wheat, and tomato [37]. The application of pure AHLs also influences plant defense responses. In Arabidopsis, 3OC14-HSL and *N*-3-oxo-dodecanyl-homoserine lactone (3OC12-HSL) treatment conferred resistance to biotrophic and hemibiotrophic pathogens, and these effects depended on strong and prolonged MPK6 activation [38]. The authors further demonstrated that 3OC14-HSL treatment followed by pathogen challenge increased phenolic compound accumulation, lignin, and callose deposition in plant cell walls. In addition, oxylipin accumulation in distal tissue, which was found to promote stomatal closure, enhanced plant resistance [28, 39]. Schenk et al. [39] noted that AHLs with short and medium side chain lengths affect root architecture development, while AHLs with long side chains induce systemic resistance in Arabidopsis. Consistent with this hypothesis, systemic resistance response was not induced when Arabidopsis roots were treated with C4-HSL or C6-HSL; instead, the expression of phytohormone-regulated genes and the content of auxin/cytokinin were altered [27]. In tomato, however, C6-HSL and, to a lesser extent, C4-HSL increased the expression of SA- and ethylene-dependent defense genes and the level of SA [29]. These contradictory findings reflect the complexity of the interactions between plants and bacteria mediated by AHLs. Nevertheless, these results indicate that bacterial AHLs can induce a primed state in plants. In this context, plant responses to different AHLs and the roles of different AHLs in priming induction warrant further investigation.

The objective of this study was to investigate whether 3OC8-HSL induces defense response priming in plants. Arabidopsis plants pretreated with 3OC8-HSL were inoculated with pathogenic bacteria, and the bacterial titer, H_2_O_2_ burst, and expression of defense-related genes were subsequently analyzed. The molecular mechanism of 3OC8-HSL-mediated priming was also assessed. We concluded that 3OC8-HSL protects Arabidopsis against a hemibiotrophic pathogen by priming the defense response and that 3OC8-HSL-mediated priming requires the SA signaling pathway.

## Results

### 3OC8-HSL protects Arabidopsis from *Pseudomonas syringae* pv. *tomato* infection

**Fig. 1:**
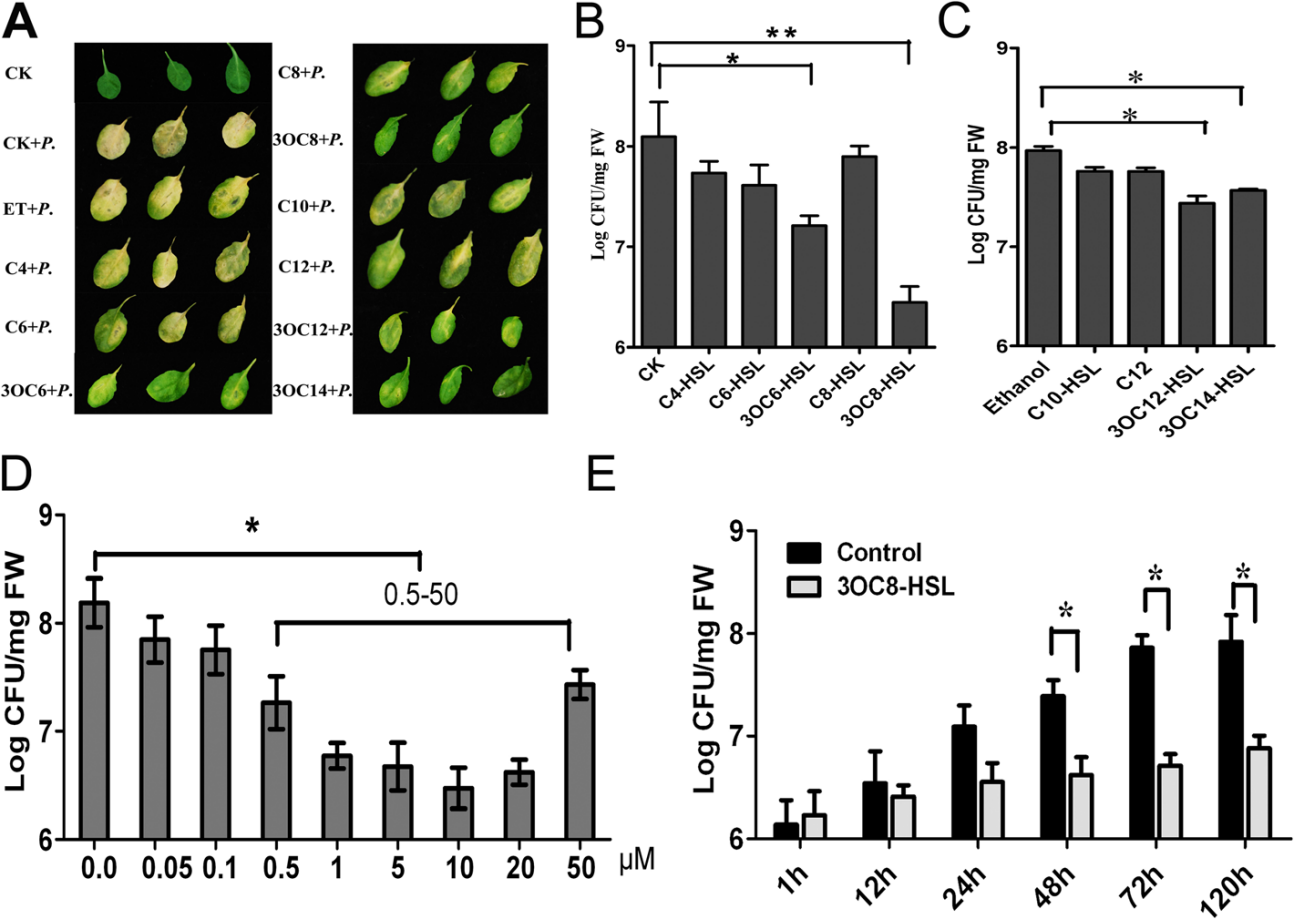
Enhanced resistance of 3OC8-HSL-treated Arabidopsis against *Pseudomonas syringae* pv. *tomato* 3000. **a**, Symptoms of *Pst*DC3000 infection on wild-type Arabidopsis pretreated with different *N*-acyl-homoserine lactone (AHL) compounds. Detached leaves from 5-week-old soil-grown Arabidopsis were pretreated with different AHL compounds at a concentration of 10 μM for 2 days prior to spray-inoculation with *Pst*DC3000 (OD_600_ = 0.1). The disease symptoms were recorded 3 days after inoculation. Abbreviations: CK, wild-type Arabidopsis Col-0 inoculated with MgCl_2_; *P.*, wild-type Arabidopsis Col-0 inoculated with *Pst*DC3000. The remaining abbreviations indicate the following pretreatments: ET, ethanol; C4, C4-HSL; C6, C6-HSL; 3OC6, 3OC6-HSL; 3OC8, 3OC8-HSL; C8, C8-HSL; C10, C10-HSL; 3OC12, 3OC12-HSL; 3OC14, 3OC14-HSL; C12, C12-HSL. **b**, Proliferation of *Pst*DC3000 in the leaves of hydroponically grown Arabidopsis plants pretreated at the roots with different 10 μM AHL (C4-C8) compounds for 48 h and with subsequent foliar spray-inoculation of *Pst*DC3000 (OD_600_ = 0.1). Colony-forming units (CFUs) were counted at 72 h post-inoculation (hpi). **c**, Proliferation of *Pst*DC3000 in the leaves of hydroponically grown Arabidopsis plants pretreated at the roots with different 10 μM AHL (C10-C1) compounds for 48 h and with subsequent foliar spray-inoculation of *Pst*DC3000 (OD_600_ = 0.1). **d**, Effect of 3OC8-HSL concentration on induction of resistance against *Pst*DC3000 in Arabidopsis. The roots of hydroponically grown plants were pretreated with different concentrations of 3OC8-HSL for 48 h prior to foliar inoculation with *Pst*DC3000 (OD_600_ = 0.1). CFUs were counted at 72 hpi. **e**, Inhibitory effect of 3OC8-HSL on *Pst*DC3000 growth in Arabidopsis. The leaves of hydroponically grown plants were inoculated with *Pst*DC3000 (OD_600_ = 0.1) after 48 h of pretreatment with 10 μM 3OC8-HSL at the roots. CFUs were counted at the indicated hpi. Data represent the average values of three independent biological replicates ± standard deviation (SD). * indicate statistically significant differences (*P* < 0.05, Student’s t test). ** indicate statistically significant differences (*P* < 0.01, Student’s t test)

3OC14-HSL and 3OC12-HSL were previously found to prime pathogen-specific defense responses in Arabidopsis and barley [38]. In contrast, resistance induction was not observed in Arabidopsis after treatment with the short-chain AHL C6-HSL [27]. Thus, tests of resistance induction by intermediate AHLs, such as *N*-octanoyl homoserine lactone (C8-HSL) and *N*-decanoyl-homoserine lactone (C10-HSL), and their derivatives can help clarify whether the response is specifically associated with long-chain AHLs and whether the oxo-group substitution at the C3 atom is necessary for this activity. Detached leaves from soil-grown Arabidopsis were pretreated with different AHL compounds (10 μM) for 2 days prior to spray-inoculation with *Pst*DC3000 (OD_600_ = 0.1). The disease symptoms were recorded 3 days after inoculation. The leaves pretreated with 3OC8-HSL, 3OC6-HSL, 3OC12-HSL, and 3OC14-HSL exhibited no visible *Pst*DC3000 symptoms, while leaves pretreated with the other AHLs turned yellow or had water-soaked lesions (Fig. 1a). We also pretreated Arabidopsis roots grown in a sterile hydroponic system with 10 μM 3OC8-HSL and eight other AHLs (with different acyl chain lengths and modifications at the C3 position) for 2 days. The plant leaves were inoculated with *Pst*DC3000 by spraying with a bacterial suspension. The bacterial colony-forming units (CFUs) in the leaf tissue were counted at 72 h post inoculation (hpi). The stock solutions of AHLs with tails of 10 carbons or more were prepared in ethanol, so ethanol pretreatment was used as the control for long-chain AHLs. Water-treated controls were used for the other AHLs tested in this study. We found that the priming effects of AHL on plant defense response depended on AHL structure. 3OC8-HSL pretreatment exerted the strongest inhibitory effect on pathogen proliferation, while 3OC6-HSL, 3OC12-HSL, and 3OC14-HSL had moderate effects on bacterial growth *in planta*. Compared to the respective controls, no significant differences in pathogen propagation were observed when plants were pretreated with C4-HSL, C6-HSL, C8-HSL, C10-HSL, and C12-HSL (Fig. 1b and c). Different concentrations of 3OC8-HSL were applied for 2 days prior to *Pst*DC3000 foliar inoculation to evaluate dose-dependent induction effects. At a concentration less than 0.5 μM, 3OC8-HSL did not decrease pathogen titer, but bacterial growth *in planta* was significantly reduced when the concentration was above 0.5 μM (Fig. 1d). To monitor the disease progression on the leaves of 3OC8-HSL-pretreated plants, we monitored CFUs for 120 h after pathogen infection. While bacterial titer gradually increased in the leaves of Arabidopsis plants without 3OC8-HSL pretreatment, pathogen proliferation was significantly inhibited in the 3OC8-HSL-pretreated plants (Fig. 1e).

The effects of 3OC8-HSL on the growth and virulence of *Pst*DC3000 were also assessed in vitro. The pathogen was grown in 3OC8-HSL-supplemented medium to a concentration of 10 μM, but it did not inhibit *Pst*DC3000 growth or affect its virulence (Additional file 3: Figure S2A and S2B). The finding that 3OC8-HSL did not directly affect the fitness or virulence of the hemibiotrophic pathogen *Pst*DC3000 in vitro although it enhanced disease perturbation *in planta* indicates that 3OC8-HSL functions as a plant defense activator.

### Pretreatment with 3OC8-HSL triggers enhanced H_2_O_2_ accumulation upon pathogen infection

**Fig. 2:**
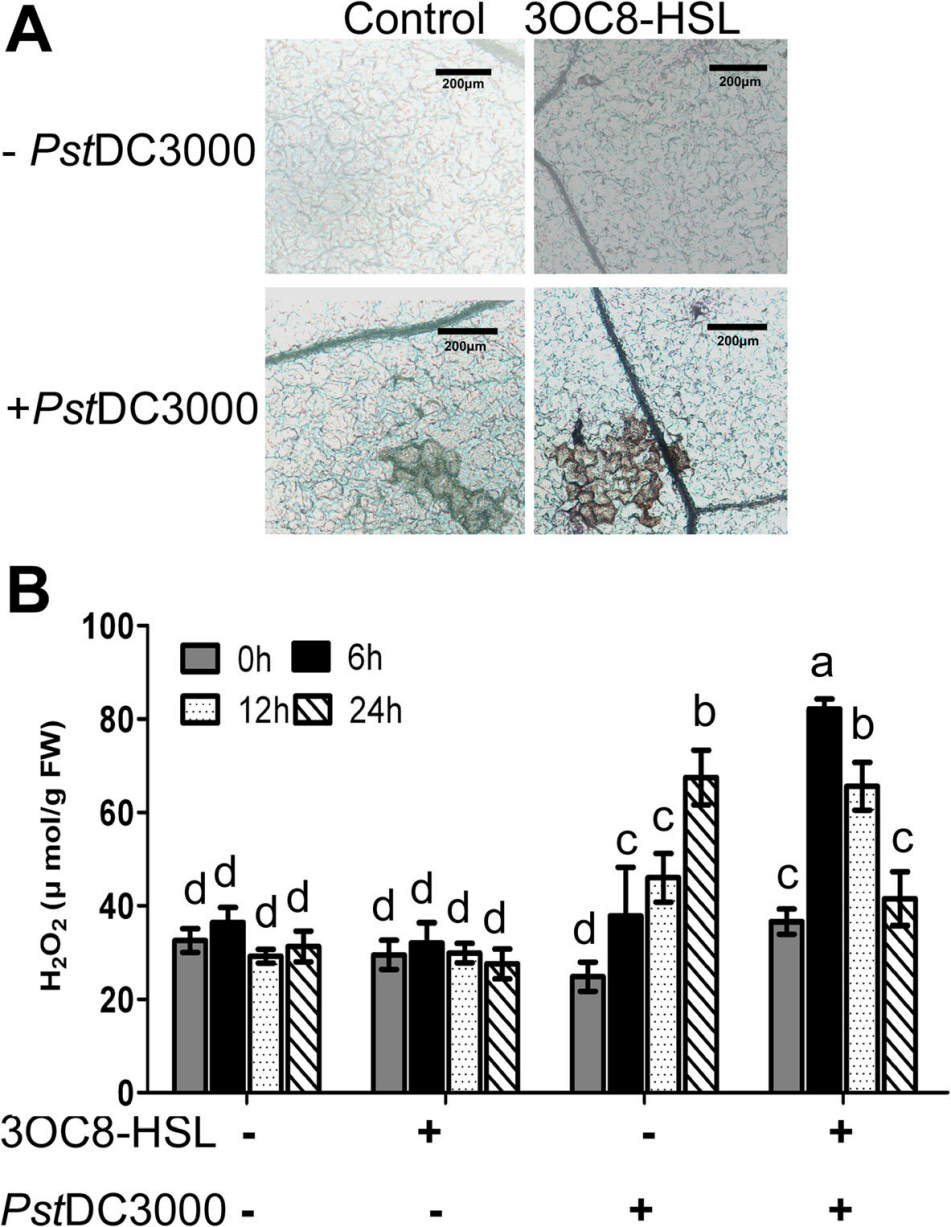
Effects of priming by 3OC8-HSL and pathogen challenge on H_2_O_2_ accumulation. **a**, Microscopic observation of H_2_O_2_ accumulation. Hydroponically grown Arabidopsis plants were pretreated in Hoagland medium containing 10 μM 3OC8-HSL or without 3OC8-HSL (control) for 2 days, then the leaves were sprayed with *Pst*DC3000 (OD_600_ = 0.1) solution and collected at 0, 6, 12, and 24 hpi. The leaves were incubated in DAB staining solution for 24 h. After de-staining, the leaves were photographed under a microscope at the indicated times (bar = 200 μm). The experiments were performed with six leaves per treatment, and similar results were obtained in three independent experiments. **b**, H_2_O_2_ quantification in Arabidopsis with 3OC8-HSL pretreatment and pathogen challenge. The samples were collected as described above, and the H_2_O_2_ content was detected according to the method described by Velikova et al. (1971). Values are means ±SD of three independent experiments. Different letters indicate statistically significant differences (P < 0.05, Duncan’s test)

When plants encounter biotic or abiotic stress, an ROS burst is triggered, which in turn activates defense responses [40]. The effect of 3OC8-HSL on H_2_O_2_ production in leaves was analyzed using the diaminobenzidine (DAB) staining method. Detached leaves of Arabidopsis Col-0 plants pretreated by adding 10 μM 3OC8-HSL to the hydroponic medium for uptake by the roots were collected after *Pst*DC3000 inoculation. The staining results for the untreated control revealed few cells with H_2_O_2_ accumulation at 6 hpi, with the deep-brown color contained in only several cells of the untreated leaves when viewed under a microscope (Fig. 2a). In contrast, a strong accumulation of H_2_O_2_ was observed after *Pst*DC3000 inoculation in 3OC8-HSL-pretreated leaves (Fig. 2a). However, 3OC8-HSL treatment alone had no effect on H_2_O_2_ production (Fig. 2a). We also measured H_2_O_2_ content in detached leaves pretreated with 10 μM 3OC8-HSL with subsequent *Pst*DC3000 infection (Fig. 2b). H_2_O_2_ formation was not detected in 3OC8-HSL-pretreated leaves without subsequent pathogen inoculation, while *Pst*DC3000 spray-inoculation of control leaves without 3OC8-HSL pretreatment caused significant H_2_O_2_ production at 24 hpi (Fig. 2b). A very rapid and strong accumulation of H_2_O_2_ was detected in 3OC8-HSL-pretreated leaves after pathogen exposure (Fig. 2b). In these inoculated leaves from pretreated plants, H_2_O_2_ accumulation peaked at 6 hpi and remained elevated until 12 hpi, suggesting that the combination of 3OC8-HSL pretreatment and *Pst*DC3000 challenge induces augmented H_2_O_2_ accumulation.

### 3OC8-HSL priming in Arabidopsis enhances PR gene expression and the activities of defense-related enzymes

**Fig. 3:**
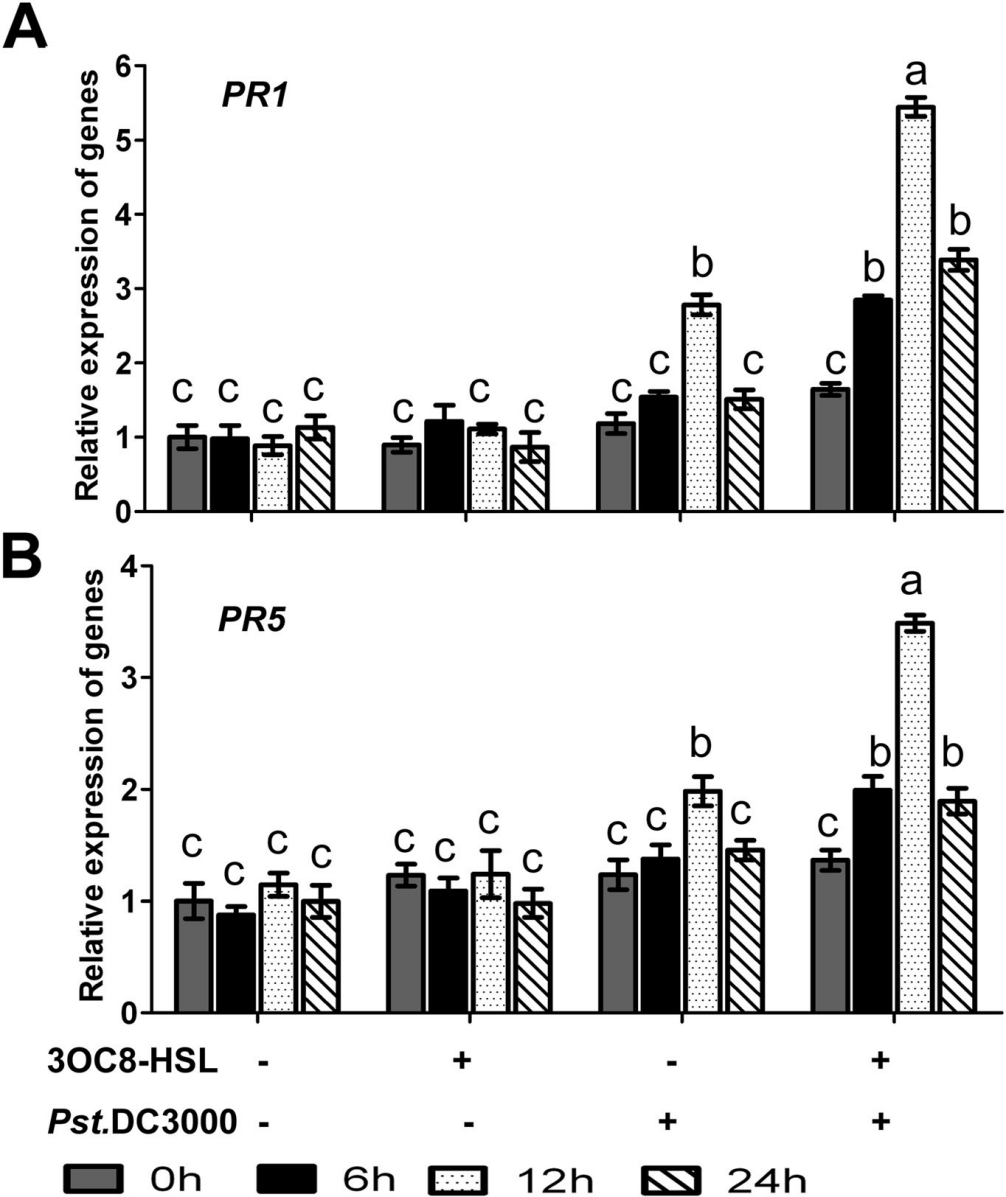
Expression of *PR1* and *PR5* in Arabidopsis plants pretreated with 3OC8-HSL and challenged with *Pst*DC3000**.** Total RNA was extracted from Arabidopsis Col-0 seedlings pretreated with 10 μM 3OC8-HSL at the roots for 48 h followed by *Pst*DC3000 inoculation. Samples were collected at the indicated time points (hpi). Real-time PCR was performed using gene-specific primers, and the relative expression levels of the induced resistance marker genes are shown. **a**, *PR1* transcript levels at the indicated hpi in response to 3OC8-HSL in Arabidopsis. **b**, *PR5* transcript levels at the indicated hpi in response to 3OC8-HSL in Arabidopsis. Values are means ±SD of three independent experiments. Different letters indicate statistically significant differences (P < 0.05, Duncan’s test)

Pathogenesis-related (PR) genes, including *PR1* and *PR5* (thaumatin-like protein), are often used as molecular indicators of the defense response against *Pst*DC3000. To investigate the kinetics of 3OC8-HSL action, *PR1* and *PR5* expression levels were monitored. In 3OC8-HSL-pretreated leaves without pathogen inoculation, *PR1* and *PR5* transcripts did not accumulate, whereas transcription of both genes was observed at 12 h after *Pst*DC3000 inoculation in Arabidopsis plants without 3OC8-HSL pretreatment (Fig. 3). In 3OC8-HSL-pretreated plants with *Pst*DC3000 inoculation, significant transcript accumulation of both genes was already detected at 6 hpi, and the levels of both genes were much higher at 12 hpi than in plants without 3OC8-HSL pretreatment (Fig. 3).

**Fig. 4:**
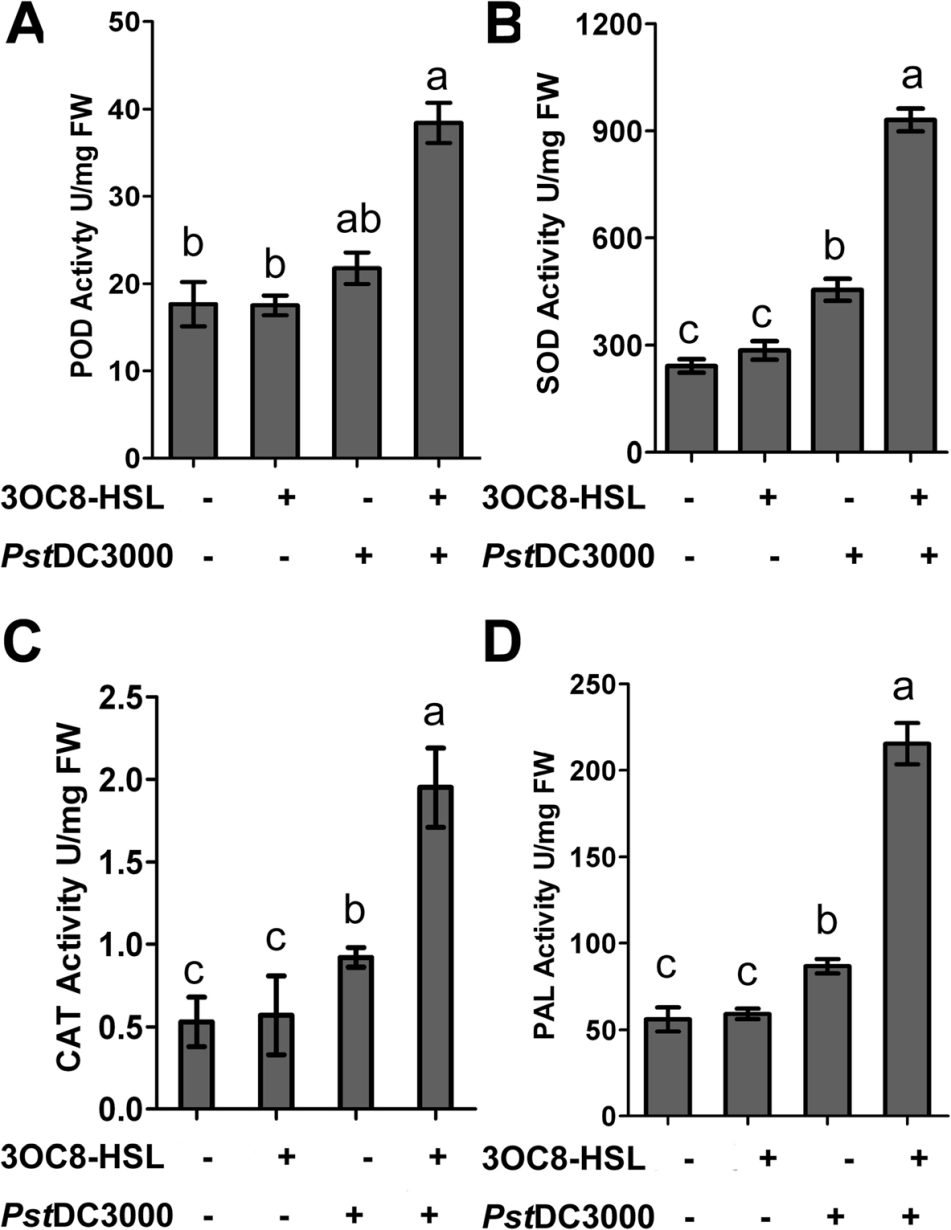
Changes in defense enzyme activities in Arabidopsis after 3OC8-HSL pretreatment and *Pst*DC3000 infection. The roots of Arabidopsis plants grown in a sterile systemic hydroponic system were treated with 10 μM 3OC8-HSL for 48 h, and the leaves were spray-inoculated with *Pst*DC3000 (OD_600_ = 0.1). The seedlings were collected at 24 hpi and frozen in liquid N_2_. The frozen tissue (100 mg) was used to detect enzyme activity. **a**, Change in POD activity. **b**, Change in CAT activity. **c**, Change in SOD activity. **d**, Change in PAL activity. Values are means ±SD of three independent experiments. Different letters indicate statistically significant differences (P < 0.05, Duncan’s test)

Defense-related enzymes such as peroxidase (POD), catalase (CAT), phenylalanine ammonia lyase (PAL), and superoxide dismutase (SOD) are key components of plant inducible defense responses. To investigate whether the effects of 3OC8-HSL on plant defense response are mediated by defense-related enzymes, 3OC8-HSL-treated and untreated plants were subjected to *Pst*DC3000 attack, and POD, CAT, PAL, and SOD activity levels were measured (Fig. 4). Without infection, 3OC8-HSL pretreatment of the roots did not induce the activity of any of the four enzymes in plant leaves relative to untreated plants (Fig. 4). On the other hand, untreated plants with *Pst*DC3000 infection had slightly increased levels of all four enzymes relative to control plants. The activity levels of all four enzymes were much more strongly induced in the leaves of 3OC8-HSL-pretreated plants with subsequent pathogen inoculation (Fig. 4). Taken together, these results suggest that 3OC8-HSL pretreatment primes augmented *PR* gene expression and activity of defense-related enzymes upon pathogen infection.

### 3OC8-HSL pretreatment potentiates SA accumulation upon pathogen infection

**Fig. 5:**
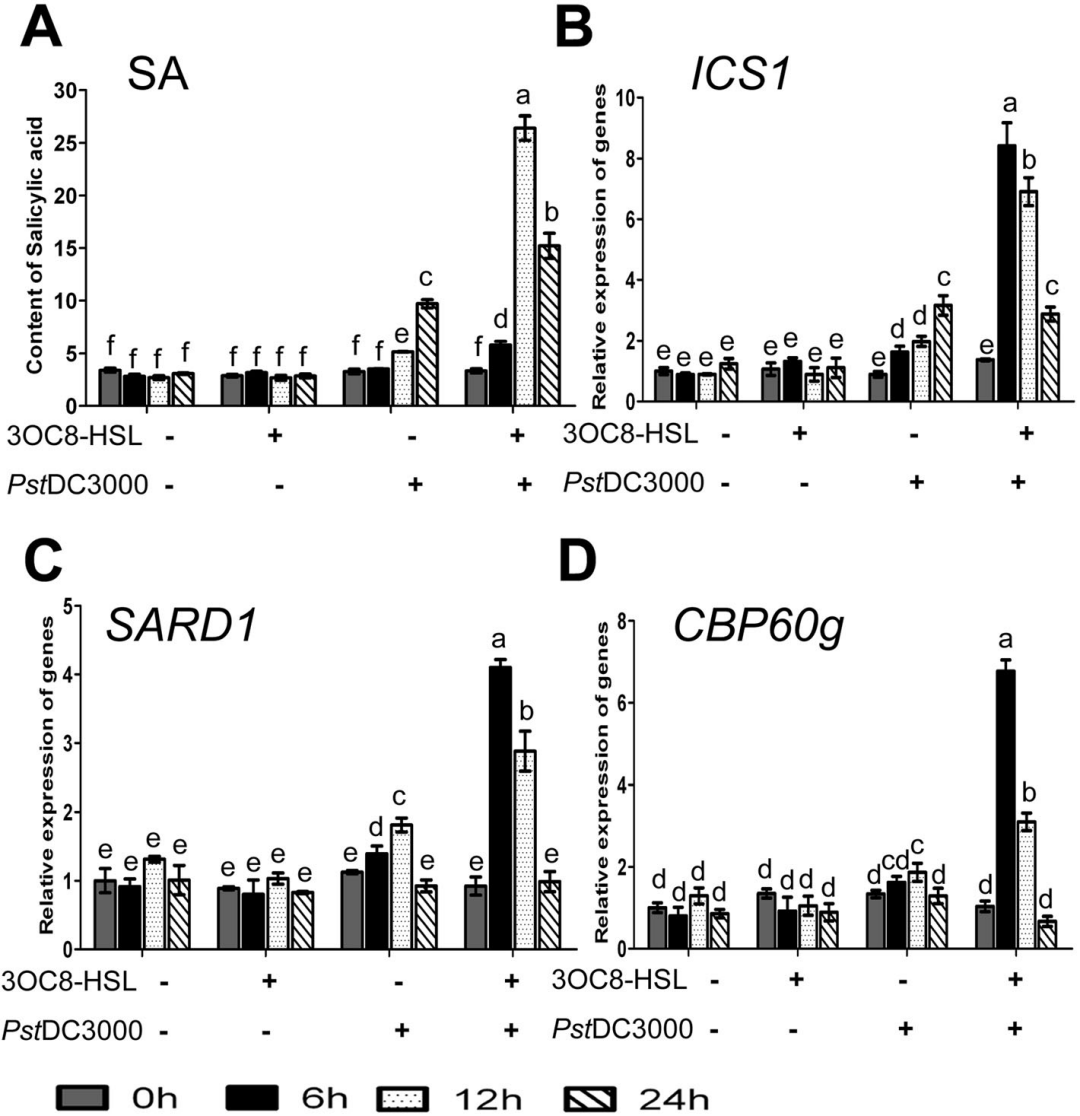
Effects of 3OC8-HSL application on SA accumulation and the expression of SA biosynthesis genes. **a**, Accumulation of free SA measured by HPLC in Arabidopsis plants after pretreatment of the roots with 10 μM 3OC8-HSL for 48 h and subsequent spray-inoculation of the leaves with *Pst*DC3000 (OD_600_ = 0.1). Leaves were harvested at the indicated times 0 h, 6 h, 12 h, 24 h. **b**-**d**, Relative expression of *ICS1*, *SARD1*, and *CBP60g* by quantitative RT-PCR using *ACTIN* for normalization. Values are means ±SD of four independent experiments. Different letters indicate statistically significant differences (P < 0.05, Duncan’s test)

The effects of 3OC8-HSL on the expression of *PR* genes, which are typically regulated by SA, indicate that SA signaling might be involved in 3OC8-HSL-induced priming in Arabidopsis. However, whether 3OC8-HSL pretreatment influences SA biosynthesis and accumulation upon pathogen inoculation remains unclear. To address this question, we measured SA levels and the expression of SA biosynthesis genes in the leaves of plants with 3OC8-HSL pretreatment of the roots for 2 days prior to *Pst*DC3000 inoculation. In 3OC8-HSL-unpretreated plants with *Pst*DC3000 infection, there was an increase in free SA content at 12 hpi to 24 hpi; in pretreated plants with infection, there was more accumulation of free SA and a much higher accumulation of free SA at 12 hpi (Fig. 5a). For both conditions, the concentration of free SA remained high at 24 hpi (Fig. 5a). These findings indicate that 3OC8-HSL primed the plants for enhanced SA accumulation in systemic tissues in response to pathogen attack.

The isochorismate (IC) pathway is the major route of SA biosynthesis in plants, and IC synthase (ICS), encoded by the gene *ICS1*, is a critical enzyme in this pathway. In addition, CBP60g and its homolog SARD1, two calmodulin (CaM)-binding transcription factors, control *ICS1* transcription [41–43] To further investigate the effects of 3OC8-HSL on the regulation of SA biosynthesis, we used qRT-PCR to quantify *ICS1*, *CBP60g*, and *SARD1* transcript levels in the leaves of wild-type Arabidopsis plants whose roots were pretreated with 3OC8-HSL for 2 days prior to *Pst*DC3000 foliar inoculation. In 3OC8-HSL-pretreated plants, *ICS1* and *SARD1* transcript levels were enhanced at 6 hpi and 12 hpi, whereas *CBP60g* transcripts were strongly induced at 6 hpi (Fig. 5b). In water-treated control plants, *Pst*DC3000 inoculation moderately increased the transcript levels of these three genes at 12 hpi. These data suggest that 3OC8-HSL potentiates the expression of SA biosynthesis genes upon pathogen attack.

### 3OC8-HSL-induced priming is dependent on the SA signaling pathway

**Fig. 6:**
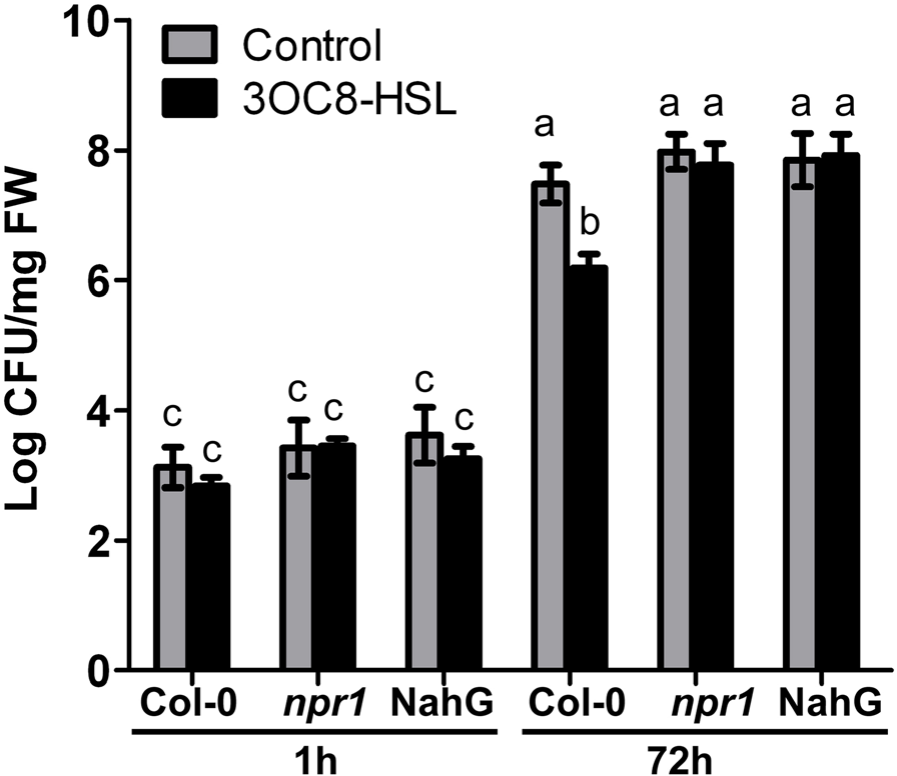
Effects of 3OC8-HSL on *Pst*DC3000 growth in wild-type Arabidopsis, *npr1–1*, and *NahG* transgenic plants. Plant were grown in a sterile systemic hydroponic system, and the roots were pretreated with 10 μM 3OC8-HSL for 48 h, followed by spray-inoculation of the leaves with *Pst*DC3000 (OD_600_ = 0.1). CFUs were counted at 1 hpi and 72 hpi. Values are means ±SD of four independent experiments. Different letters indicate statistically significant differences (P < 0.05, Duncan’s test)

The findings showed that the expression of SA-responsive genes was primed by 3OC8-HSL upon infection with the hemibiotrophic pathogen *Pst*DC3000 (Fig. 3). To further investigate the dependency of 3OC8-HSL-enhanced resistance on SA signaling, we applied 3OC8-HSL to the bottom-layer medium of the following plants grown in a sterile systemic hydroponic system prior to *Pst*DC3000 infection: wild-type Arabidopsis Col-0, the *NPR1*-deficient mutant *npr1–1*, and *NahG* transgenic plants (*NahG*). All plants were grown for 2 weeks in hydroponic culture then pretreated with 3OC8-HSL at the roots for 2 days prior to *Pst*DC3000 foliar inoculation. In wild-type plants 3 days after inoculation, 3OC8-HSL pretreatment significantly reduced the bacterial titer *in planta* compared to the water-treated control (Fig. 6). In contrast, 3OC8-HSL-enhanced resistance to *Pst*DC3000 was impaired in *npr1–1* and *NahG* plants (Fig. 6), suggesting that the SA-dependent pathway is required for 3OC8-HSL priming of the resistance response in Arabidopsis.

## Discussions

In this study of the impact of 3OC8-HSL on the plant immune system, we demonstrated that 3OC8-HSL, which is known to promote primary root elongation [32, 44], can also prime plants for defense responses against hemibiotrophic bacteria. 3OC8-HSL pretreatment augmented H_2_O_2_ production, primed the expression of defense-related genes, and potentiated the activity of defense-related enzymes in plants upon bacterial pathogen challenge. Moreover, our data suggest that the SA-related signaling pathway is required for 3OC8-HSL-primed resistance.

AHLs vary in terms of acyl chain length (4 to 18 carbons) and in the substitution at theγposition of the fatty acid chain with hydroxyl (OH) or oxo (O) groups, and numerous AHLs have been identified from over 70 species of Gram-negative bacteria [45]. Plants respond to different AHLs in different ways. Generally, AHLs with short side chains (4 to 6 carbons) regulate root growth and development, while long-chain AHLs such as C12- and C14-HSL induce plant resistance [27, 28, 30, 32, 38, 39, 44, 46–48]. Direct evidence has been lacking for the involvement of intermediate AHLs, such as C8-HSL and C10-HSL, and their substitution with oxo-groups at the C3 position in the fatty acid chain in plant defense response. In the present study, we showed that 3OC8-HSL pretreatment enhanced resistance against *Pst*DC3000 in Arabidopsis. In vitro analysis revealed that 3OC8-HSL did not directly arrest growth of the bacterial pathogen or affect its virulence. Thus, 3OC8-HSL had no direct effect on the causal pathogen but enhanced disease resistance *in planta*, indicating that 3OC8-HSL acts instead as a plant defense activator. We previously reported that 3OC8-HSL can promote primary root growth in Arabidopsis [32]. It is reported that 3OC8-HSL is the common quorum sensing signal in natural habitat [49]. Several plant pathogenic bacteria in rhizosphere including *Pseudomonas syringae*, *Pectobacteria* and *Pantoea* produce 3OC8-HSL as signal molecule for cell-cell communication [50]. Plant may evolve the mechanism during the long course of interaction between plant and microbe that sense the AHL to recognize the presence of invading bacteria in surrounding environment and take action to defence themselves. Considered together with the present results, we conclude that 3OC8-HSL enhances root growth and also acts as a priming agent in plants. In addition, our data imply that substitution by an oxo-group at the C3 position in AHLs is associated with more efficient resistance induction, as 3OC8-HSL and 3OC6-HSL primed defense reactions in Arabidopsis while C8-HSL and C6-HSL did not (Fig. 1). Similarly, Schikora et al. [38] observed the strongest effect on resistance induction in plants pretreated with 3OC14-HSL and 3OC12-HSL.

ROS production, such as H_2_O_2_ generation, is one of the earliest events in the plant defense response against pathogen attack. 3OC8-HSL pretreatment prior to pathogen challenge potentiated H_2_O_2_ accumulation; in contrast, *Pst*DC3000 inoculation without prior pretreatment led to slower and moderate H_2_O_2_ production in Arabidopsis leaves (Fig. 2). Thus, rapid and strong H_2_O_2_ synthesis is one of the defense mechanisms primed by 3OC8-HSL, consistent with previous reports that identified enhanced H_2_O_2_ accumulation after pathogen inoculation as one of the typical responses of primed plants [51, 52]. In addition, pathogen challenge after 3OC8-HSL treatment led to enhanced activity of POD, SOD, and CAT relative to untreated controls (Fig. 4). These enzymes are involved in H_2_O_2_ metabolism in plant cells, so the findings suggest that the mechanisms maintaining H_2_O_2_ homeostasis were also primed by 3OC8-HSL. Taken together, these data clearly demonstrate that H_2_O_2_ accumulation is a component of 3OC8-HSL-induced priming.

SA plays a critical role in Arabidopsis resistance against *P. syringae* [7]. We demonstrated the involvement of the SA signaling pathway in 3OC8-HSL-induced priming of resistance against *Pst*DC3000 through several lines of evidence. Namely, *Pst*DC3000 infection following 3OC8-HSL pretreatment led to the following: 1) enhanced accumulation of free SA in the leaves; 2) strong and rapid expression induction of the SA biosynthesis gene *ICS1* along with *CBP60g* and *SARD1*, two transcription factor genes that regulate *ICS1*; and 3) potentiated expression of *PR1* and *PR5*, two genes typically regulated by the SA pathway upon pathogen infection. In addition to these findings, 3OC8-HSL-primed resistance against *Pst*DC3000 was attenuated in the *npr1–1* mutant and *NahG* transgenic plants. 3OC8-HSL pretreatment without subsequent pathogen challenge did not induce SA accumulation or the expression of *ICS1*, *CBP60g*, *SARD1*, *PR1*, or *PR5*. Although *Pst*DC3000 inoculation without pretreatment triggered SA biosynthesis and *PR1* and *PR5* expression, the peak occurrence time and the magnitude of induction were much later and lower than those in 3OC8-HSL-pretreated plants. These findings suggest that 3OC8-HSL pretreatment induces a state of extreme sensitivity to pathogen challenge, allowing plants to rapidly and strongly initiate SA signaling to prevent or mitigate pathogen attack. Similar SA dependency has also been reported in BABA-treated tobacco [53] and Arabidopsis [54]. Similarly, 3OC14-HSL-induced resistance depends on an oxylipin and the SA signaling pathway [28, 47]. Oxylipins, including JA and related metabolites, are lipid-derived signaling compounds that accumulate in response to pathogen infection. However, 3OC14-HSL-induced resistance was found to be JA-independent [28]. Further investigation is required to determine whether phytohormones other than SA, such as JA, are involved in 3OC8-HSL-induced resistance.

Joshi et al. [55] recently reported that plant phenolic acids, including cinnamic acid (CA) and SA, affect the virulence of *Pectobacterium aroidearum* and *P. carotovorum subsp. brasiliense* via quorum sensing (QS) regulation [55]. Exposing bacteria to a nonlethal dose of SA inhibited the expression of QS genes, including *expI*, *expR*, and *PC1–1142* (*luxR* transcriptional regulator), and down-regulated the expression of virulence factors, such as *pecS*, *pel*, *peh*, and *yheO*, which are regulated by the QS system. Accordingly, SA treatment reduced the virulence of *Pectobacterium spp.* in potato and calla lily. Considering the present results, 3OC8-HSL could trigger SA cascades and thereby potentially affect the pathogenic bacteria.

Strengthening of the cell wall upon pathogen attack provides an additional structural barrier against pathogen invasion [56, 57]. Increased callose deposition, phenolic compounds, and lignin were shown in flg22-challenged, AHL-primed Arabidopsis [28]. In C10-HSL-treated barley shoots, Götz-Rösch et al. [57] found that dehydroascorbate reductase activity increased 384% relative to control plants, whereas SOD activity in barley roots decreased to 23% of that in control plants upon C6-HSL treatment. Plant growth and pigment contents in barley and yam bean showed only small responses to three different AHLs (C6-HSL, C8-HSL, and C10-HSL), indicating that AHL treatment triggered tissue- and compound-specific changes in the activities of important detoxification enzymes. PAL is the key enzyme for the synthesis of precursors required for lignification and cell wall strengthening [58]. PAL activity is an extremely sensitive indicator of stress conditions and is commonly associated with defense responses [59]. In this study, we observed an enhanced elevation of PAL activity after 3OC8-HSL application to roots followed by foliar inoculation with the bacterial pathogen. POD and SOD are oxidoreductive enzymes that participate in wall-building processes, and the activities of both enzymes were significantly enhanced by 3OC8-HSL pretreatment of Arabidopsis plants prior to pathogen infection. Collectively, our results suggest that 3OC8-HSL primes plant resistance to bacterial pathogens via cell wall reinforcement, similar to the effect of 3OC14-HSL.

## Conclusions

Our results demonstrate a novel biological function for 3OC8-HSL: it confers disease resistance through the priming of plant defense response, leading to a restriction of pathogen growth *in planta* and suppressed propagation of the inoculum. 3OC8-HSL shifts the plant into a highly competent state and triggers a fortified molecular and cellular defense response upon subsequent pathogen challenge. Priming by AHLs in plants may represent an effective and economical pathogen response strategy with minimal metabolic costs because the metabolic requirements of the priming mechanism itself are relatively low. In the context of conventional disease control methods, 3OC8-HSL could serve as a novel priming agent that satisfies environmental regulations.

## Methods

### Plant growth, AHL pretreatment, and pathogen inoculation

Seeds of *Arabidopsis thaliana* ecotype Columbia-0 (Col-0), Col-0 expressing the bacterial *NahG* gene, and the T-DNA insertion null mutant *npr1–1* (CS3726) were obtained from the Arabidopsis Information Resource (TAIR, https://www.arabidopsis.org). Seeds were surface sterilized with 75% (v/v) ethanol for 1 min and 30% (v/v) NaClO for 5 min. After five washes with sterile distilled water, the seeds were germinated and grown on agar plates containing MS medium (pH 5.8). The plants were placed in a growth chamber with a 16 h light/8 h dark photoperiod, 100 μmol m^− 2^ s^− 1^ light intensity, and a temperature of 22 ± 2 °C. For pathogen proliferation, H_2_O_2_ accumulation, enzyme activity, and transcriptional and biochemical analysis, the plants were cultivated in a sterile systemic hydroponic system to eliminate the effects of unknown microbes on the plants (Additional file 2: Figure S1). This system physically separates the roots and shoots of plants. After 10 days of germination on MS agar plates, seedlings with two leaves and a root length of 2 cm were transplanted into a sterile plastic container (a repurposed 18 cm × 11 cm Eppendorf holder covered with Parafilm) with 450 ml sterile Hoagland medium then cultivated for 2 weeks. After this period, the bottom-layer medium was exchanged with fresh medium. AHL pretreatment was performed by directly adding AHLs into the medium. Two days later, the leaves were spray-inoculated with *Pst*DC3000. For the disease symptom assay following *Pst*DC3000 infection, detached leaves from soil-grown Arabidopsis were used. The 10-day-old plate-grown seedlings described above were transplanted into a steam-sterilized soil mixture of commercial potting soil/perlite (3:1) then cultivated in a growth chamber for 4 weeks. The detached leaves were floated on sterile half-strength MS medium, treated with AHLs for 2 days, then spray-inoculated with *Pst*DC3000.

The nine AHLs used for pretreatment in this study (C4-HSL, C6-HSL, 6-HSL, C8-HSL, 3OC8-HSL, C10-HSL, C12-HSL, 3OC12-HSL, and 3OC14-HSL) were purchased from Sigma-Aldrich (Taufkirchen, Germany). C4-HSL, C6-HSL, 6-HSL, C8-HSL, and 3OC8-HSL are water-soluble; C10-HSL, C12-HSL, 3OC12-HSL, and 3OC14-HSL are soluble in ethanol. Thus, plants treated with water (non-pretreated) were used as the control for the five water-soluble AHLs, and plants treated with ethanol were used as the control for the four ethanol-soluble AHLs. AHLs were dissolved in their respective solvents as 10 mM stock solutions and used at working concentrations of 10 μM or as indicated. All compound solutions were sterilized by passing them through a 0.22 μm filter. AHLs were added directly into Hoagland medium and mixed well. *Pst*DC3000 was cultured overnight in King’s B medium until OD600 = 0.6–1.0 with rifampicin (50 μg/ml). The bacterial cells were collected by centrifugation, washed in 10 mM MgCl_2_, and resuspended in 10 mM MgCl_2_. To inoculate Arabidopsis with *Pst*DC3000, the bacterial suspension was adjusted to OD_600_ = 0.1 in 10 mM MgCl_2_ with 0.02% Silwet 77. Two days after AHL treatment, the plants were spray-inoculated with the bacterial suspension until all leaves were covered with fine droplets. At the indicated time points after pathogen inoculation, 100 mg leaf tissue was harvested and homogenized in 10 mM MgCl_2_. Serial dilutions of the homogenate were plated onto King’s B media plates containing selective antibiotics for colony-forming unit (CFU) counting with 50 μg/mL rifampicin. All experiments were performed with the untreated control plants. Three independent biological experiments were conducted with three technical replicates each.

### Analysis of H_2_O_2_ accumulation

To determine H_2_O_2_ accumulation after priming by 3OC8-HSL, the hydroponically grown Arabidopsis plants were pretreated in Hoagland medium containing 10 μM 3OC8-HSL or without 3OC8-HSL (control) for 2 days. The leaves were sprayed with *Pst*DC3000 (OD_600_ = 0.1) solution. ROS formation was detected using the DAB (Sigma-Aldrich, Germany) staining method at 6 h post-inoculation (hpi). The leaf samples were incubated in DAB solution (1 mg/ml 3,3-diaminobenzidine in water) overnight at 22 °C and de-stained in ethanol/chloroform/trichloroacetic acid (4:1:0.15) for 24 h. The leaves were photographed using a Leica DM4000B microscope (Leica Co., Germany). The experiments were performed three times with six leaves for each treatment. H_2_O_2_ content was determined according to the method based on the oxidation of Fe^2+^ in the presence of oxylanol orange, which yields a colored complex with Fe^3+^ at A560 [60]. H_2_O_2_ content in the samples was determined by comparison to the standard curve. Three independent biological experiments were conducted with three technical replicates each.

### Transcriptional analysis

As indicated above, hydroponically grown Arabidopsis above-ground seedlings with or without 3OC8-HSL pretreatment were collected at 0, 6, 12, and 24 h after spray-inoculation of the aerial leaves with *Pst*DC3000 (OD_600_ = 0.1). Total RNA of the homogenized plant tissues was extracted using TaKaRa RNA Plus reagent (Dalian, China) For relative quantification of gene expression, the comparative CT method [61] and the 7500 Real Time PCR System (Applied Biosystems, Foster City, CA, USA) were used. The target gene level was normalized to the endogenous reference gene β*-Actin*. Each data point represents the average of three independent experiments. As a technical control, each qRT-PCR experiment was repeated four times on the same 96-well plate. qRT-PCR was performed using the primers listed in Additional file 1: Table S1.

### Salicylic acid measurement

Extraction and quantification of free salicylic acid (SA) were performed using the leaves of 3OC8-HSL-pretreated hydroponically grown Arabidopsis seedlings 0, 6, 12, 24 h after *Pst*DC3000 inoculation of the leaves. Plant tissues were frozen and ground in liquid N_2_. The frozen tissue (0.5 mg) was used for SA extraction and quantification via HPLC. Chromatography was performed on a Waters 1525 HPLC system (Waters Technologies). Separation was achieved on an Inertsil ODS C_18_ column (50 3 4.6 mm, 2.5 mm; GL Sciences). Sodium acetate (90%) in water and methanol (10%) were employed as mobile phases A and B, respectively. For fluorescence intensity detection, the excitation wavelength was 313 nm, and the emission wavelength was 405 nm [62]. The flow rate was 0.5 ml/min, and the injection volume was 10 μl. The column temperature was maintained at 25 °C.

### Determination of enzyme activity

The leaves of 3OC8-HSL-pretreated hydroponically grown Arabidopsis seedlings were collected 24 h after *Pst*DC3000 inoculation (OD_600_ = 0.1). Frozen tissue (100 mg) was used for detection of enzymes activity. SOD activity was measured according to Beauchamp and Fridovich method [63]. The method based on CAT decomposition of H_2_O_2_ can be quickly terminated by adding ammonium molybdate. The remaining H_2_O_2_ interacts with ammonium molybdate to produce a kind of yellow complex. H_2_O_2_ was added to the extracted supernatant as the substrate, and the production amount was measured at 240 nm to calculate CAT activity [64]. POD activity measurements were performed according to Wang et al. [65], based on the principle of the POD-catalyzed H_2_O_2_ reaction. For the enzyme activity measurement, the absorbance wavelength was 420 nm. PAL enzyme activity was determined as described previously [66]. Based on the principle of PAL-catalyzed phenylalanine ammonia formation of trans-cinnamic acid, enzyme activity was determined by measuring the absorbance at 290 nm. Three independent biological experiments were conducted with three technical replicates each.

### Statistical analysis

For all experiments, the data were statistically analyzed using the DPS v7.05 program. Student’s test were used in Fig. 1. Univariate and multivariate analyses (ANOVA) with a Duncan’s new multiple range tests were used in Fig. 2, 3, 4, 5, 6. All data were represented as means ±SD of three-four independent experiments.

## Supplementary information

**Additional file 1 : Table S1.** Primer information of genes investigated in qRT-PCR.

**Additional file 2 : Figure S1**. Hydroponic systems.

**Additional file 3 : Figure S2**. 3OC8-HSL has no direct effect on *Pst*DC3000 growth and its virulence.

## Data Availability

The datasets used and/or analyzed during the current study are available from the corresponding author on reasonable request.

## Abbreviations

3OC8-HSL: *N*-3-oxo-octanoyl-homoserine lactone
ABA: Abscisic acid
AHL: *N*-acyl-homoserine
BTH: Benzothiadiazole
CAT: Catalase
CFU: Colony-forming unit
ETI: Effector-triggered immunity
GSH: Glutathione
H_2_O_2_: Hydrogen peroxide
HR: Hypersensitive response
INA: 2.6-dichloro-isonicotinic acid
ISR: Induced systemic resistance
MAMP: Micro-associated molecular patterns
MeJA: Methyl Jasmonate
PAL: Phenylalanine ammonia lyase
PAMP: Pathogen-associated molecular patterns
POD: Peroxidase
PR: the Pathogenesis-related genes
PRRs: Pattern recognition receptors
*Pst*DC3000: *Pseudomonas syringae* pv. *tomato* DC3000
PTI: Pattern-Triggered Immunity
QS: Quorum sensing
ROS: Reactive oxygen species
RT-qPCR: Quantitative real-time polymerase chain reaction
SA: Salicylic acid
SOD: Superoxide anion radicals
SAR: Systemic acquired resistance
TTSS: Type III secretion system
