# Supplementary material for: N-3-oxo-octanoyl-homoserine lactone-mediated priming of resistance to Pseudomonas syringae requires the salicylic acid signaling pathway in Arabidopsis thaliana
Source: BMC Plant Biol. 2020 Jan 28;20:38. doi: 10.1186/s12870-019-2228-6 (PMC6986161; doi:10.1186/s12870-019-2228-6)
Supplement: Supplementary file 1 — Additional file 1 : Table S1. Primer information of genes investigated in qRT-PCR. [file 12870_2019_2228_MOESM1_ESM.pdf]

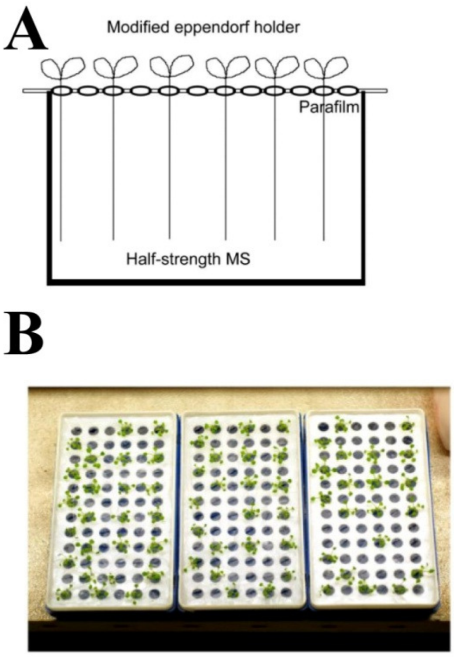

Supplementary Figure 1. Hydroponic system. This system allows the separation of roots and shoots. Roots, grown in the lower layer, were treated with AHLs, and the upper leaves were inoculated with bacteria, preventing direct contact between the roots and bacteria. Seeds were surface sterilized with 75% (v/v) ethanol for 1 min and 30% (v/v) NaClO for 5 min. After five washes with sterile distilled water, the seeds were germinated and grown on agar plates containing MS medium (pH 5.8). Plants were placed in a growth chamber with a 16 h light/8 h dark photoperiod, 100 μmol $m^{-2}s^{-1}$ light intensity, and a temperature of 22±2°C. When the seedlings had two leaves and their roots reached 2 cm in length, they were transplanted into a sterile plastic basin (a repurposed 18 cm x 11 cm Eppendorf holder covered with Parafilm) containing 450 ml of half-strength MS medium. The medium was changed every three days. AHLs were added directly into the medium.
