# Supplementary material for: N-3-oxo-octanoyl-homoserine lactone-mediated priming of resistance to Pseudomonas syringae requires the salicylic acid signaling pathway in Arabidopsis thaliana
Source: BMC Plant Biol. 2020 Jan 28;20:38. doi: 10.1186/s12870-019-2228-6 (PMC6986161; doi:10.1186/s12870-019-2228-6)
Supplement: Supplementary file 2 — Additional file 2 : Figure S1. Hydroponic systems. [file 12870_2019_2228_MOESM2_ESM.pdf]

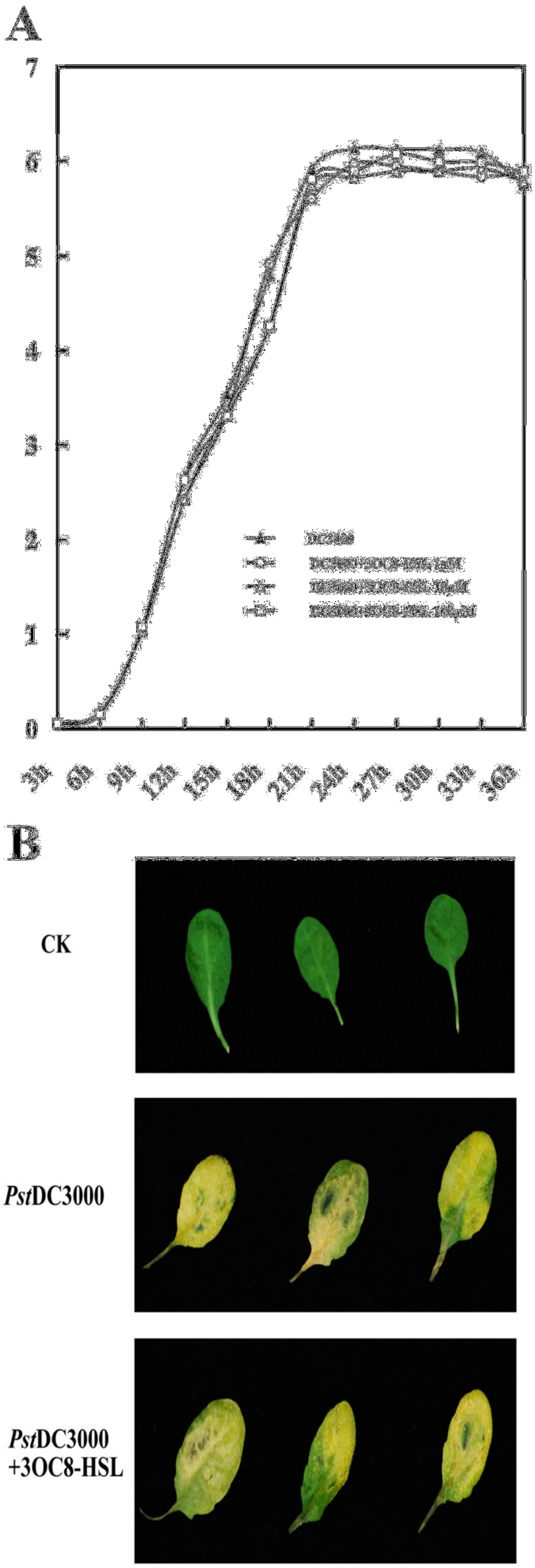

A
7
6
5
4
3
2
1
0
3h
6h
9h
12h
15h
18h
21h
24h
27h
30h
33h
36h
B
CK
*Pst*DC3000
*Pst*DC3000
+3OC8-HSL

Supplementary Figure 2. 3OC8-HSL has no direct effect on *Pst*DC3000 growth and its virulence. A. *Pst*DC3000 was cultivated in medium containing different concentrations of 3OC8-HSL, and OD_{600} population density was measured at different times. The results demonstrated that 3OC8-HSL did not inhibit the growth of *Pst*DC3000. B. Detached Arabidopsis leaves were inoculated with *Pst*DC3000 grown in medium with or without 10 μM 3OC8-HSL, and the disease symptoms were observed at 48 hpi. The results indicated that the presence of 3OC8-HSL in the bacterial growth medium had no impact on bacterial virulence. Abbreviations: CK, wild-type Arabidopsis Col-0 inoculated with $MgCl_2$; *Pst*DC3000, wild-type Arabidopsis Col-0 inoculated with *Pst*DC3000 cultivated in KB medium without 3OC8-HSL; *Pst*DC3000+3OC8-HSL, wild-type Arabidopsis Col-0 inoculated with *Pst*DC3000 cultivated in KB medium supplemented with 3OC8-HSL.
