# Supplementary material for: N-3-oxo-octanoyl-homoserine lactone-mediated priming of resistance to Pseudomonas syringae requires the salicylic acid signaling pathway in Arabidopsis thaliana
Source: BMC Plant Biol. 2020 Jan 28;20:38. doi: 10.1186/s12870-019-2228-6 (PMC6986161; doi:10.1186/s12870-019-2228-6)
Supplement: Supplementary file 3 — Additional file 3 : Figure S2. 3OC8-HSL has no direct effect on PstDC3000 growth and its virulence. [file 12870_2019_2228_MOESM3_ESM.pdf]

Supplementary Table1 Primer information of genes investigated in qRT-PCR

Primer	Sense	Antisense
ICS1	5′-GAACTCAAATCTCAACCTCC-3 ′	5′-ACTGCGACGAG AGAAGAAAC-3′
SARD1	5'-GGAATGTCTGATAGAAAGTGGGAAGT-3'	5'-ACGTAGGGCTGGTTGAGGGA-3'
CBP60g	5'-GTTCTCGTCTTCTCGGGTCG-3'	5'-CATCACCGTTAGGTCTCCAGT-3'
Actin2/8	5'-CCAGAAGGATGCATATGTTGGTGA-3'	5'-GAGGAGCCTCGGTAAGAAGA-3'
PR1	5'-GTGCCAAAGTGAGGTGTAACAA-3'	5'-CGTGTGTATGCATGATCACATC-3'
PR5	5'-ATGTGAGCCTCGTAGATGGTTAC-3'	5'-GATCCATGACCTTAAGCATGTCG-3'

qRT-PCR was done using primers listed in the table.
